# Supplementary material for: Exploiting ecology in drug pulse sequences in favour of population reduction
Source: PLoS Comput Biol. 2017 Sep 28;13(9):e1005747. doi: 10.1371/journal.pcbi.1005747 (PMC5643144; doi:10.1371/journal.pcbi.1005747)
Supplement: S1 Text — It includes phase trajectories for different values of skewness s, and a brief discussion of how nmin and the population composition would be affected if the the total antibiotic load τ + tr is fixed. (PDF) [file pcbi.1005747.s001.pdf]

Exploiting ecology in drug pulse sequences in favour of population reduction: Supporting information

M. Bauer[1], I. R. Graf[1], V. Ngampruetikorn[2], G. Stephens[2,3], E. Frey[1*],

1 Arnold-Sommerfeld-Center for Theoretical Physics and Center for NanoScience, Department of Physics, Ludwig-Maximilians-Universität München, D–80333 Munich, Germany
2 Biological Physics Theory Unit, Okinawa Institute of Science and Technology Graduate University, Onna, Okinawa 904 0495, Japan
3 Department of Physics & Astronomy, Vrije Universiteit Amsterdam, 1081HV Amsterdam, The Netherlands

*frey@lmu.de

1. Fig. 1 of the main text for different τ

In order to gauge how different N-pulse sequences compare for different τ, and whether Fig. 1 in the main part of the text changes much, we show the corresponding figures for $\tau = 30$ and $\tau = 90$ in Fig. A a and b in S1 Text, respectively. We see that for smaller τ, the region where the higher N-sequences get better increases. This occurs especially for negative s, where the region where the final pulses of the higher N-sequences are best (bottom right corner, marked with black line in Fig. 1 of the main text) extends towards much smaller t_r, and merges with the region of the bottom left, an intermediate pulse in the higher N-pulse sequence is best. The extension towards smaller t_r of the region where the last pulse is best can be understood by an argument similar to the main text discussed around Fig. 4 d-e, where one gauges what $t_w^{(f)}$ is required for the first pulse to be better; since τ is smaller, the t_r where $t_r = \tau - t_w^{(f)} \equiv \tau - t_o^{\text{final}}$ is smaller too. For larger τ, the opposite is the case for the bottom right corner, for exactly the same reason. The regions where the first pulse of longer sequences are best moves down towards more negative s, but also shrinks in t_r compared to $\tau = 60$, as for $\tau = 90$ the $t_r^{(1)}$ of the single pulse is here already much longer than $t_o + t_r^{(N)}$ (see the main text). However, the regions do not change qualitatively. This might of course be different if one considers $\tau < t_o$ or $\tau < t_o^{\text{final}}$, but this would go beyond the scope of this work.

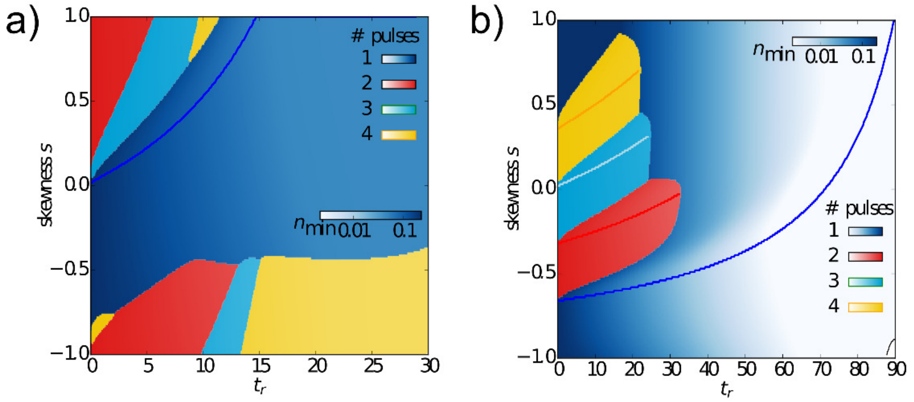

Fig A. The main figure of the paper, Fig. 1, for different τ: $\tau = 30$ for the left (a) and $\tau = 90$ for the right panel (b), respectively. We see that the regions where the pulse sequences are better shift consistently: the region where the higher pulses of the sequences yield the lowest global minima (bottom right) shifts towards the left for increasing τ, while the region where the first pulse of the sequence is better than the single pulse (top left) moves inwards and out. In general, for larger τ, the single pulse does better for a larger area in (s, t_r) space.

2. Fig. 1 of the main text for different cost for the resistant species, switching rates and death rates

In the main text, we chose a fitness cost of $k = 0.1$ for the growth rate of the more resistant species, since this parameter gave a good, yet not too confusing overview over where which pulse sequence yielded the best results. In Fig. B, we show the analogous figures to Fig. 1 in the main text for two different costs $k = 0.4$ and $k = 0.01$ (top panels, (a) and (b)). These different costs change the values for the optimal initial and final times spent in the *low* environment, t_o, and t_o^{final} (see section 3 of the supporting information).

The general features of Fig. 1 remain the same: the single pulse is best for the largest part of the phase diagram and is lowest at the thick blue line, where the skewness is such that the initial time in the *low* environment matches the t_o for this parameter combination. The first pulse of a longer pulse sequence is best in the top left corner, around the corresponding thick line where the initial time in the *low* environment for this first pulse matches t_o. The region marked with the black curve corresponds to the region where the last pulse of these higher order pulse sequences is best. Compared to Fig. 1 in the main text, the increased cost of $k = 0.4$ (Fig. Ba in S1 Text) leads to an increased importance of this last region. In addition, the regions where intermediate pulses of the longer pulse sequences are best (marked with white lines), are also larger than in Fig. 1 of the main text, but occur at exactly the predicted positions (in the bottom right corner, and between the regions where first pulses of longer sequences yield lowest minima, in the top left).

For the smaller cost of $k = 0.01$ (Fig. Bb) compared to $k = 0.1$ in Fig. 1 in the main text, the behaviour is analogous to what we have described so far: the regions encircled in white disappear, and region where the last pulse of the longer sequences yield lower minima (marked with the black line) becomes smaller. In addition, similarly to what happens for longer τ (see Fig. F in S1 Text), the importance of the region where the single pulse yields best results increases. This is due to the fact that the optimal time spent initially in the *low* environment t_o is shorter for shorter costs, and that thus τ/t_o increases. Thus means that the t_r of the single pulse is longer, and can reduce the

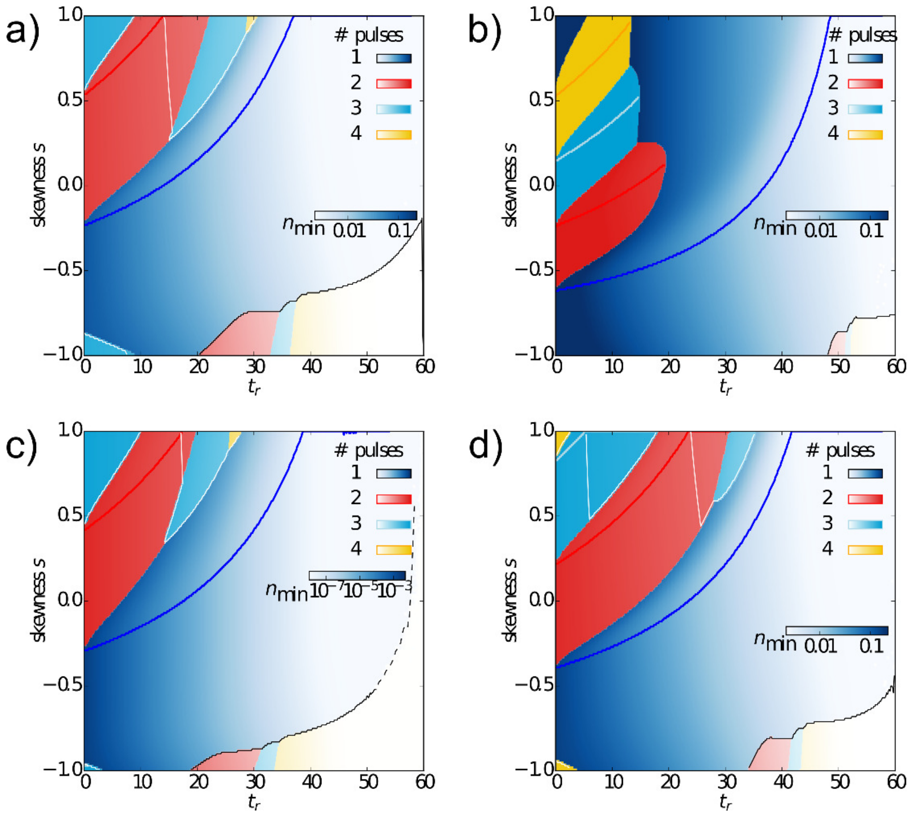

Fig B. The main figure of this work, Fig. 1, for different rates and costs for mutation: a) $k = 0.4, \delta = 0.1$; b) $k = 0.01, \delta = 0.1$; c) $k = 0.4, \delta = 0.3$; (so far for all $\mu_w = 10^{-6}, \mu_r = 0$, like in the main text) d) $\mu_w = 10^{-7}, \mu_r = 10^{-4}$ and $\delta = 0.1, k = 0.1$ like in the main text. The general features are independent of the system parameters. For the largest part of skewnesses and time in the *high* environment, including the region where the skewness is such that initial time in the *low* environment matches the optimal initial time spent in the *low* environment, t_o, for the corresponding parameters (thick blue lines), the single pulse is best. The first pulse of a higher order pulse sequence is best when the initial time in the *low* environment of this first pulse matches t_o, and in areas around it (top left corner). The last pulse of such higher sequences is best for large t_r and negative skewnesses (marked by a black line). The dominant differences between the different figures are the exact boundaries of these regions, and the regions where intermediate pulses of the longer pulse sequences yield the lowest minimum (white lines). Due to the high decrease in population for the high death rate of $\delta = 0.4$, we could not precisely resolve the exact position of the black boundary and thus marked it with dashes.

population for longer, than $t_o + t_r/N$ for the Nth pulse sequence.

In addition, the changed costs influence the absolute value of the minimal population at the best pulse. The minimal population then is smaller for higher fitness costs, as the population decays more strongly due to the slower growth of the more resistant species.

Finally, we also investigate a combination of increased death rate and cost (Fig. Bc, to be compared with the Fig. Ba above for the same cost), as well as different switching rates. Again, the features of the figures are exactly the same as in Fig. 1 of the main text. The increased death rate meant that the population reduction was higher, and so for high t_r the different pulse sequences gave very small and effectively indistinguishable lowest minima. Thus, we could not resolve the region where the last pulse of the longer sequences was best precisely, and marked it with a dashed black line in these cases.

Changing the switching rates (Fig. Bd) also leads to only very small changes of general features compared to Fig. 1 in the main text. We note the increase in the areas marked with the white lines, where intermediate pulses of higher pulse sequences do best, but again at positions where they would have occurred for Fig. 1. Thus, we conclude that the main features of, as well as the relevant explanations for Fig. 1 in the main text, are robust against parameter variations.

3. Optimal $t^{(i)}_{w,\mathrm{optimal}} = t_o$ and $t^{(f)}_{w,\mathrm{optimal}} = t^{\mathrm{final}}_o$ for different parameters

In Fig. C we show how the optimal initial and final times in the *low* environment can change with respect to system parameters.

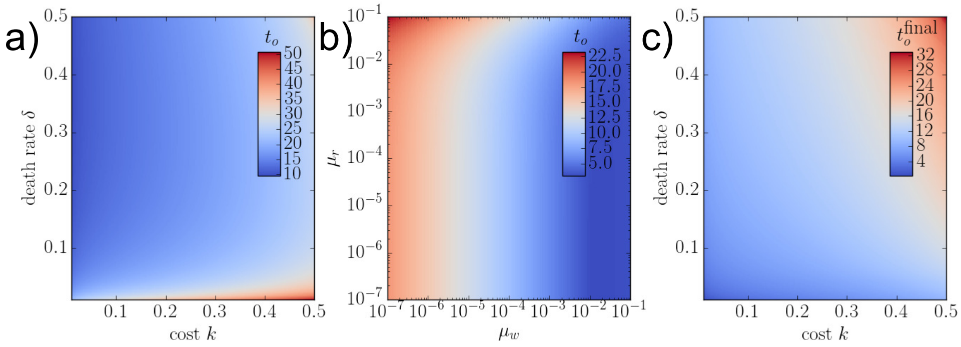

Fig C. Left panel (a): t_o as a function of death rate δ and cost k in the growth rate for the more resistant species. For small δ and large k, t_0 can take values of up to $\approx 40/\lambda$, and is thus an important parameter. Middle panel (b): t_o as a function of the switching rates μ_r and μ_w, which determine only the initial condition given by the attractive fixed point of the *(free)* regime. There is hardly any dependence on μ_r for $\mu_r \ll \delta$, while t_o decays with increasing μ_w which implies more resistant agents in the system initially; Right panel (c): Limit of $t_o^{\mathrm{final}}(t_w^{(i)} = 0)$ for large t_r as a function of death rate δ and cost k. This saturating value of t_o^{final} increases considerably towards large δ and large k and is always smaller or equal than t_0.

Panel (a) of Fig. C shows the variation of t_o as a function of death rate δ and cost k in the growth rate of the resistant species, $k = \lambda - \lambda_r$. Obviously, t_o increases when this cost increases, as the resistant species will take longer to grow. For fixed cost, there is a minimal t_o at a certain death rate, with t_o increasing towards both small and large death rates. This is due to the fact that for small growth rate costs for the resistant species, the death rate dominates for the wild type which decays faster, while for large growth rate costs, the resistant species' growth is also drastically limited by the high

death rate. This behaviour is most pronounced for large cost $k = 0.5$, where t_o diverges for $\delta = 0.5$, since then r cannot grow any more at all. In general, we see that t_o can acquire high high values of around $t_o \approx 50$. This means that the optimal $t_w^{(i)}$ can have a very large effect, and explain why sometimes more moderate treatments are functional, even though they come with the risk of evolving resistance in principle.

In Fig. C b we show how t_o depends on the switching rates μ_r and μ_w. Even if those rates do not change the actual dynamics very much, they have a big influence on t_o, as they crucially influence the initial condition, that is the attractive fixed point of the *(free)* environment. For larger μ_w the population starts out with a relatively higher amount of resistant species and less wild-type so that the resistant species can take over the population faster, thus decreasing t_o. We note that t_o decreases linearly with μ_w for μ_w up to $\mu_w \approx 10^{-2}$. The dependence on μ_r is less pronounced for small μ_w and μ_r as to lowest order in $\mu_{w,r}$ the fixed point of the free-system only depends on μ_w.

Concerning t_o^{final}, we note that it depends on the preceding $t_w^{(i)}$ and t_r. It will be maximal when $t_w^{(i)} = 0$, as then r has not yet grown while w has been depleted, and thus if $t_w^{(i)} = 0$ and $t_r = 0$, $t_o^{\text{final}} = t_o$. For $t_w^{(i)} = 0$, t_o^{final} decays from this t_o as a function of t_r, and saturates at a certain value for high t_r, which again depends on the system parameters. In Fig. C c we show the value t_o^{final} saturates to for high t_r. We do not show its dependence on $t_w^{(i)}$, as then we would have to additionally satisfy the constraint that $t_w^{(i)} + t_r + t_o^{\text{final}} = \tau$, which would distract from the value of t_o^{final}. As such, t_o^{final} can take any value between Fig. C a ($t_r = 0$) and Fig. C c (large t_r).

4. Comparing minima within a pulse sequence

There are two competing concepts that determine during which pulse the population minimum could occur: First, the time during which the population effectively decays exponentially, which can be larger than t_r for pulses early in the sequence where w is still present, and will eventually be t_r for pulses late in the sequence; and second, the initial population, from which the decay leading to the lowest minimum starts. The latter might be lowest in intermediate pulses, where w has decayed, but r has not yet taken over the population.

We want to discuss at which t_r a second pulse can yield a lower minimum than a first pulse for $s = 1$ and $s = -1$.

For $s = 1$, we have seen that the maximal period during which the population decays is $t_w^{(i)} + t_r$ for $t_w^{(i)} \leq t_o$ [in principle if $t_w^{(i)} + t_r$ is small, there could be another contribution from $t_w^{(i),2ndpulse}$, that is a contribution like $t_w^{(f)}$ only at the beginning of the 2nd pulse], which is maximal when $t_w^{(i)} = t_o$. Since pulses are periodic, and since we consider $\tau < 126$ (which effectively is a requirement on t_r being such that the population will be lower than the minimum of the *(low)* regime (obtained after t_o) after the second $t_w^{(i)}$ regime), n will grow up to r^{low} in the following *(low)* regime of the second pulse; there, the population can only decay during t_r, and thus the second pulse will have a higher minimum for $t_w^{(i)} = t_o$. More precisely, for pulses with $t_w^{(i)} > t_o$, the period of effective population decay in the first pulse shortens, and will correspond to $\max(t_o, t_r)$ for pulses with $t_w^{(i)} > t_o + t_{regrowth}$, where $t_{regrowth}$ in our case corresponds to approximately 10. In the *(low)* regime of the second pulse, the population will definitely increase if $t_w^{(i)} > t_o$, meaning that in the second pulse, the effective decay period will only be t_r. The decay period of the second pulse is thus either equally long as during the first pulse (t_r), or shorter when $t_r < t_o$, with the starting population of decay being definitely at r^{low}. In the latter case, the second pulse will definitely have a higher minimum; in the first, where $t_w^{(i)} > t_o + t_{regrowth}$ and $t_r > t_o$ and the decay periods of both pulses are effectively the same, the second pulse will have an identical

minimum to the first pulse. (Also, this latter case only applies for sequences with $\tau_(N) > 2 * t_o + t_{regrowth} \approx 35$). Thus, the second pulse has a higher or equal minimum whenever $t_w^{(i)} > t_o$.

For $s = -1$, we already noted one can have a very low minimum in the second pulse if the population decays consistently for $t_r^{(N)} + t_w^{(f),(N)} + t_r^{(N)}$. However, the second pulse will actually yield a lower minimum even for larger $t_w^{(f),(N)}$: unless $t_w^{(f),(N)}$ is larger than the time r needs to grow up to its fixed point r^{low}, the effective decay will be longer than $t_r^{(N)} + t_o^{\text{final}}$. We can estimate at which t_r we expect the $n(t = t_r) = w^*_{(iii)} e^{-\delta\tau} + r^*_{(iii)} e^{-\delta\tau} e^{\lambda_r t^{\text{off}}}$ to be lower than $n^*_{(iii)}$: this occurs for $t_w^{(f)} < (1/\lambda_r)\ln(n^*_{(iii)}/m^*_{(iii)}) + \tau\delta/\lambda_r$, which corresponds to $t^{\text{off}} < 16$ for the sequence with two pulses where $2\tau^{(N=2)} = \tau = 60$, and thus $2t_r^{(N=2)} > 32$. Indeed, Fig. 1 shows that for $t_r > 40$ the sequence with two pulses performs better than the single pulse, as here the second pulse of the two pulse sequence is lower.

For this t_r, a small increase in s from $s = -1$ implies the presence of the $t_w^{(i),(N)}$, which means that r regrows faster during the $t_w^{(f),(N)}$ phase, as well as a shorter $t_w^{(f),(N)}$. Thus, the first pulse in the sequence will yield a lower minimum again, and then certainly the single pulse is better.

5. Phase space trajectories (Fig. 5 a) for skewnesses $s = -0.5$, $s = 0.2$ and $s = 0.5$

In Fig. 5 a we show phase space trajectories for pulse sequences with $\tau = 60$, $t_r = 10$ and skewness $s = 0.9$, consisting of one, two or three pulses, respectively. The trajectories start at the *(free)* fixed point close to the w-axis and evolve towards the r-axis. r/w increases from pulse to pulse since the more resistant species r steadily takes over the population. Furthermore, the population composition at the end of the pulse sequence is similar for all three trajectories. The same holds true for other skewnesses. In Fig. D we show exemplary phase space trajectories for skewnesses $s = -0.5$, $s = 0.2$ and $s = 0.5$.

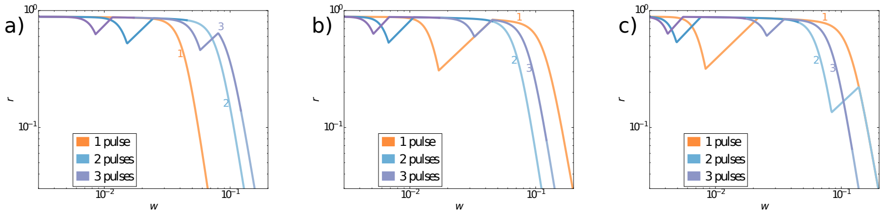

Fig D. Phase space trajectories for pulse sequences with $\tau = 60$ and $t_r = 10$, consisting of one, two or three pulses, respectively. Three different skewnesses are considered: $s = -0.5$ (a), $s = 0.2$ (b) and $s = 0.5$ (c). As in Fig. 5 a the trajectories start at the *(free)* fixed point close to the w-axis (beyond the limits of the figure) and r/w steadily increases from pulse to pulse. Furthermore, the population composition at the end of the pulse sequences is similar for one, two or three pulses.

6. Constant amount of antibiotics: Estimate

As shown in Fig. E, keeping $t_{\text{sum}} = \tau + t_r$ constant shows that the dependency on the skewness is much less pronounced than for keeping τ constant, which we explain in the following.

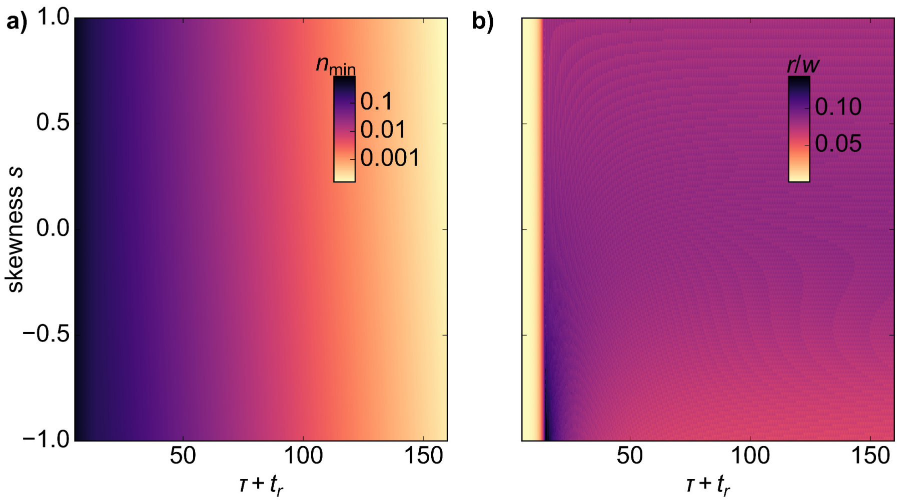

Fig E. Population minimum $n_{\min}$ (a) and composition r/w (b) for a single pulse of fixed $\tau + t_r$ instead of constant τ and fixed t_r. Both quantities show little dependency on the skewness s. For this case, there are two competing effects: the smaller t_r, the less the resistant species is depleted, but simultaneously the smaller t_r the larger τ and thus, the wild-type is depleted longer. As a result, not only optimising the onset but also avoiding a large offset is beneficial.

For the case of fixed t_{sum}, there are two competing effects: on the one hand, for small t_r, $\tau = t_{\mathrm{sum}} - t_r$ is larger than for large t_r, and thus for small t_r the population w can be depleted longer. On the other hand, larger t_r increase the chance to reduce the resistant species r, and thus also n. Thus, for $s = 1$, where the pulse starts with the *(low)* regime (see Fig. 2 in the main text), it makes sense to exploit the full optimal onset time, such that $\tau = t_r + t_o$, since then r has not grown much until the beginning of the *(high)* regime. This then means that $t_{\mathrm{sum}} = 2\tau - t_o$, and thus $\tau \approx t_{\mathrm{sum}}/2 + 7.7$. On the contrary, for $s = -1$ when the pulse starts with the *(high)* regime, it is best to use $t_w^{(f)} \approx t_o^{\mathrm{final}}(t_w^{(i)} = 0, \text{large } t_r)$ and so $t_r \approx \tau - t_o^{\mathrm{final}}(t_w^{(i)} = 0, \text{large } t_r)$ in order to prevent the resistant species from taking over the population in the subsequent *(low)* regime. So, it is ideal to use $\tau \approx t_{\mathrm{sum}}/2 + t_o^{\mathrm{final}}(t_w^{(i)} = 0, \text{large } t_r)/2 \approx t_{\mathrm{sum}}/2$. Together, these two strategies mean the pulses are optimised in such a way that their durations do not differ drastically for the different skewnesses. Thus, the best minimum and the mutant/wild-type ratio is approximately the same for all skewnesses with a little improvement (deterioration) towards skewness s = 1 for the minimum (ratio). This is also reflected in the ratio r/w in FigEb, which sharply increases until it makes sense to use $t_r > 0$, namely at $\tau + t_r = t_o$. For low s, the ratio r/w is slightly higher, as the *(low)* regime where the resistant species can grow, is at the end of the pulse. For higher values of $\tau + t_r$, the ratio is very similar across all skewnesses.
